# At-Line Characterization of Droplet Size Distributions Using a Simple, Voltage-Based Sensor for Continuous Production of Dense Oil in Water Emulsions

**DOI:** 10.1021/acs.iecr.4c03979

**Published:** 2025-02-07

**Authors:** Akshay Ravi, Amol V. Ganjare, Vivek V. Ranade

**Affiliations:** Multiphase Reactors and Intensification Group Bernal Institute, University of Limerick, Limerick V94T9PX, Ireland

## Abstract

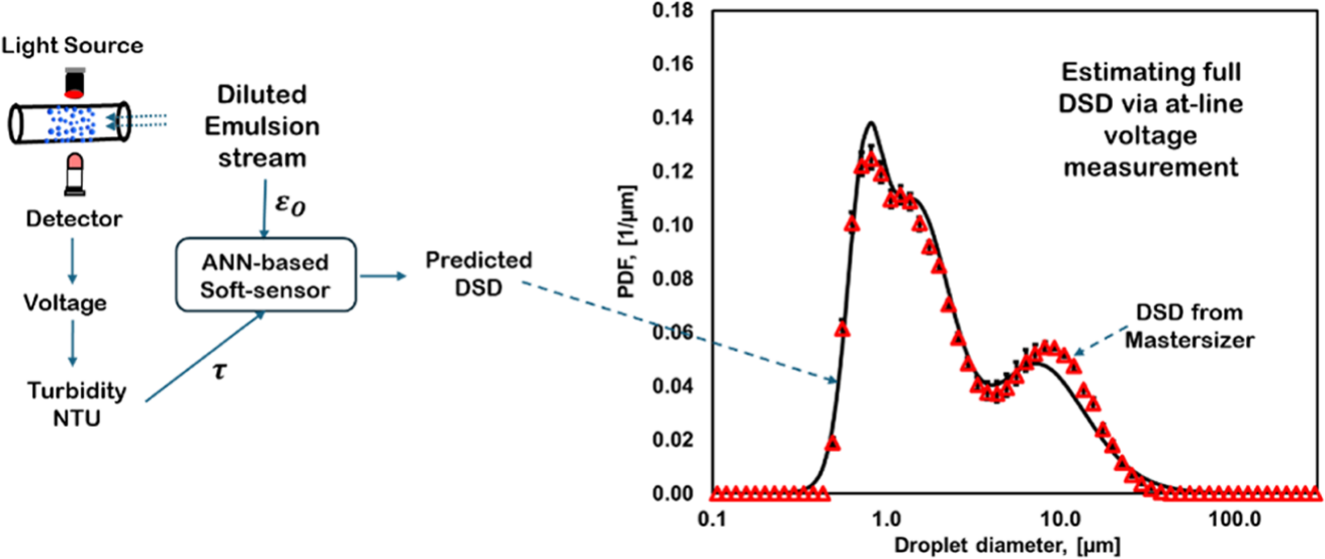

There is a growing need for real-time characterization of droplet size distribution (DSD) for the continuously produced emulsions. In this study, we developed an at-line DSD characterization method using a turbidity-based soft sensor and demonstrated its application for continuously generated oil in water emulsions using a vortex-based hydrodynamic cavitation device. The concept of using an off-line turbidity meter and an ANN-based soft sensor for estimating DSD was recently demonstrated in our previous study. In this study, we further developed this concept for real-time characterization of DSD using an inexpensive, at-line turbidity meter providing an output in terms of voltage. Combined with the previously developed ANN-based soft sensor, the at-line voltage measurements were shown to be useful for the estimation of DSD and characteristic diameters. The emulsions of rapeseed oil (RO) in water with oil volume fractions of 0.15, 0.30, and 0.45 were produced in a continuous mode. Vortex-based cavitation device was used in a loop configuration with a key operating parameter being the ratio of the circulating flow rate through the loop (*Q*) and the net flow rate of emulsion (*q*_net_). The influence of the *Q*/*q*_net_ ratio and volume fraction of oil on DSD, Sauter mean diameter (*d*_32_), other characteristic diameters, and droplet breakage efficiency (η) was investigated. The at-line turbidity measurements and ANN-based soft sensor were able to estimate the Sauter mean diameter within ±10% for oil volume fraction up to 0.45 and *Q*/*q*_net_ ratio up to 100. The developed methodology and results will be very useful for realizing decentralized and continuous emulsion production with at-line DSD measurements.

## 1 Introduction

Emulsions are essential in various sectors of industry^1−3^ such as personal care (moisturizer, body lotion, etc.), food industry (cheese, butter, ice cream), and pharmaceuticals (lotion, ointments). In all of these sectors, there is an increasing trend toward custom-made and tailored products.^3^ The primary goal of this is to provide such tailored products to customers in a distributed manner, reducing the steps needed to create ready-to-use products. The shift toward on-demand production of customized emulsions necessitates the development of innovative methods for producing emulsions with desired critical quality attributes (CQAs) on demand. Despite the long-standing use of emulsions, significant gaps remain in our understanding and our ability to produce the desired emulsions on demand. Extensive work is being undertaken to produce emulsions with the desired droplet size distribution (DSD) in a continuous mode of operation, and equipment and devices are being developed.^4^ Various methods and equipment for emulsion preparation are available, including high-pressure homogenizers, microfluidics, rotor-stator systems, ultrasonication, membranes, and others.^5^ These techniques are classified according to their energy input into high-energy methods (including high-pressure homogenization, colloid mills, ultrasonication, and microfluidics) and low-energy methods such as emulsion inversion point and phase inversion temperature.^6^ Emerging emulsion technologies include acoustic and hydrodynamic cavitation, irradiation, high-hydrostatic pressure, microwave, pulsed electric field, and ohmic heating.^7^ One of the most promising technologies for producing emulsions is hydrodynamic cavitation.^1^

Hydrodynamic cavitation (HC) involves generating, growing, and collapsing vapor cavities in liquids. HC is achieved by creating low-pressure zones (close to the liquid’s vapor pressure at the operating temperature) where cavities form. As these cavities move to higher-pressure areas and experience pressure fluctuations, under certain conditions, they implode, producing intense shear and localized hot spots.^8^ The intense localized shear generated by cavitation can be used to produce fine emulsions.^1,8^ Recently, Gode et al.^4^ showed the continuous mode of emulsion production using a vortex-based hydrodynamic cavitation device. In that work, emulsification devices along different scales of vortex-based hydrodynamic cavitation devices were compared based on the energy efficiency of emulsification (η), energy consumption per kilogram of emulsion (*E*), interfacial area unit energy consumption (*A*_*net*_)_*P*_, Sauter mean diameter (*d*_32_), other characteristic diameters (such as *D*10, *D*50, and *D*90), and droplet size distribution (DSD). The characterization of DSD was conducted using off-line analysis methods. The work was, however, restricted to low oil volume fractions (∼5% by volume). It is important to evaluate the application of HC in the continuous production of dense oil in water emulsions (15–60% oil in water). In this study, we have used a setup similar to that used by Gode et al.^4^ for producing continuous emulsions. The goal was to develop and evaluate methods for generating dense oil in water emulsions and real-time characterization of DSDs as a first step toward controlling DSD in a continuous emulsions production process.

The droplet size distribution (DSD) is a critical quality attribute (CQA) that significantly influences the rheology, appearance, and stability. Understanding and controlling DSD are therefore crucial in the production of high-quality emulsions. The task of real-time or at-line measurement of DSD is not straightforward and often requires expensive techniques.^9^ Typical process analytical tools (PAT) used for online particle characterization such as particle vision and measurement (PVM) and focused beam reflectance measurements (FBRM) have limitations in terms of characterizing small (∼10°μm) droplets. These techniques are also significantly expensive and their deployment for decentralized emulsion production is not feasible.^10^ The study by Unnikrishnan et al.^11^ presents an automated approach for evaluating the quality of emulsions in pharmaceutical manufacturing using an image-based technique, which is implemented through a principal component-based discriminant analysis (PC-DA) model on acquired micrographs of emulsions using a microscope. In another work, Unnikrishnan^12^ used machine learning-based models to process acquired images using an image segmentation approach. Despite the recent advances in the conventional and machine learning-based image processing methodologies, the DSD estimations from imaging systems still face challenges in edge detection, handling overlapping droplets and dense emulsions and real-time analysis.^13−15^ The image-based techniques also face issues related to the limitations on resolution and visualization and are often expensive and not suitable for distributed, on-demand manufacturing. It is therefore essential to develop an inexpensive and robust method for at-line characterization of DSDs which is capable of characterizing fine emulsions containing at least some fraction of droplets in a submicron range.

Recently, Upadhyay et al.^16^ have shown that off-line turbidity and UV absorbance measurements can be used for estimating characteristic diameters (such as Sauter mean diameter, D10, D90) of dense oil in water emulsions. Using the experimental data of DSDs and off-line measurements of turbidity, recently Ranade and Ranade^9^ have developed and validated a method for the estimation of full DSD using a single measurement of turbidity (in terms of NTU) at a known value of oil volume fraction using machine learning (artificial neural network [ANN]). In this study, we used these concepts and developed a method for at-line characterization of DSDs for continuously produced dense oil in water emulsions. The method is based on a commercially available, inexpensive sensor that can be conveniently fitted at-line. The appropriate methodology for using the voltage acquired from such sensor to estimate DSD was established using a previously developed soft sensor. The methodology was developed and evaluated for continuous production of rapeseed oil (RO) in water emulsion production for oil volume fractions (α_O_) of 0.15, 0.30, and 0.45. The loop configuration^4^ was used with four different ratios of circulation flow (*Q*) and net flow of oil and water (*q*_net_= *q*_w_+ *q*_o_) as 1, 5, 20, and 100. It was shown that the developed methodology based on at-line measurements of voltage after appropriate dilution and the soft sensor was successful in estimating full DSDs of produced emulsions. We hope that this study on the at-line and real-time characterization of DSDs will stimulate further research on the on-demand production of emulsions with tailored DSDs.

## 2 Experimental Section

### 2.1 Experimental Setup and Procedure

**Figure 1:**
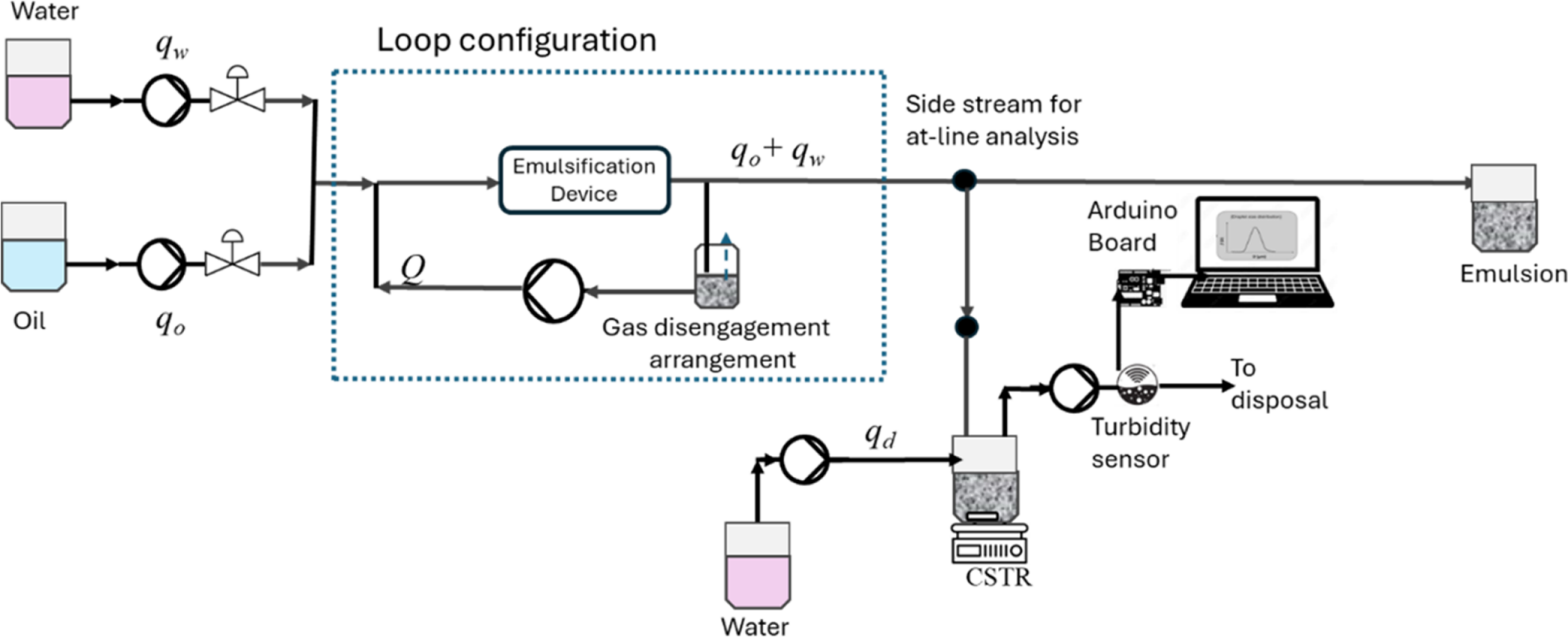
Schematic of continuous and at-line characterization experimental setup.

In this study, an experimental setup was designed to generate continuous emulsions of rapeseed oil (RO) in water emulsions using a vortex-based hydrodynamic cavitation device (VD). The design and arrangement of experimental setup is shown schematically in Figure 1 (a photograph of the setup is shown in Figure S1 of the Supporting Information [SI]). As can be seen from Figure 1, the setup consists of two feed tanks containing DI water (with surfactant) and RO. Two separate peristaltic pumps (longer pump WT600–2J and BT100–3J) were used for feedwater and RO streams with flow rates of *q*_w_ and *q*_o_, respectively. These two streams were mixed using a T junction (sourced from RS pro, PTFE, 8 mm ID). The combined stream of oil and water is introduced into the flow loop containing a cavitation device. The flow in this loop is circulated by a peristaltic pump (longer pump WT600–2J). A gas disengagement vessel is included in the flow loop (see Figure 1). The outlet stream containing product emulsion is drawn from this flow loop toward a product storage vessel.

A side-stream from the product outlet stream is taken out for at-line characterization. This side-stream of emulsion (with a flow rate of *q*_e_) is collected in a separate holding tank (with a volume of 150 mL, stirred by a magnetic stirrer of size 3 cm at 300 rpm, which was sourced from Fisher Scientific). For facilitating at-line characterization using the turbidity sensor, a diluent stream (surfactant containing DI water) was fed to this holding tank with a flow rate of *q*_d_. A separate peristaltic pump was used to maintain the level in the holding tank by pumping out diluted emulsion with a flow rate of (*q*_d_ + *q*_e_). A turbidity sensor (sourced from DFROBOT Gravity) was installed on this flow line. The sensor was operated in analog mode and the voltage output from the turbidity sensor was acquired via Arduino board (UNO R3 model) with an acquisition frequency of 2 Hz to laptop. The acquired raw voltage data was processed without any filters or amplifiers as discussed in Section 2.2. In this study, a vortex-based hydrodynamic cavitation device (VD) was used. The device had a characteristic throat diameter (*d*_T_) of 3 mm and a nominal flow rate of 1 LPM. More details of device geometry are provided by Simpson and Ranade.^17^

The rapeseed oil (RO) (ρ_O_ = 915 kg/m^3^, μ_O_ = 6.2 mPa·s, sourced from Newgrange Gold, Tesco, Ireland) and DI water (ρ_W_ = 997 kg/m^3^, μ_W_ = 0.8 mPa·s, sourced from Elga ultrapure water system) mixed with 2% (w/v) surfactant TWEEN 20 (sourced from MP Biomedicals, LLC, France) were used for producing oil in water emulsions. Previous work of Thaker and Ranade^10^ had shown that 2% (w/v) surfactant of TWEEN 20 was adequate to prevent droplet coalescence. The oil and water flow rates were selected in such a way to produce emulsions with oil volume fractions (α_O_) of 0.15, 0.30, and 0.45 . The circulation flow rate through the loop was set to 1340 mL/min, which offered a pressure drop across the cavitation device as 200 kPa. The net flow rate of emulsions, *q*_net_ (*q*_O_+*q*_W_), was then selected to achieve the values of *Q*/*q*_net_ as 1, 5, 20, and 100. The operating parameters used in the experiments are given in Table 1.

**Table 1:** Details on the Experiments Performed to Produce Emulsions with Recirculation Flow Rate *Q* = 1340  and Temperature: ∼ 22°C

oil volume fraction, α_o_	*Q*/*q*_net_	RO flow rate, *q*_oil_ [mL/min]	water flow rate, *q*_water_ [mL/min]	net flow rate, *q*_net_ [mL/min]
0.15	1	201	1139	1340
	5	40.2	227.8	268
	20	10.05	56.95	67
	100	2.01	11.39	13.4
0.30	1	402	938	1340
	5	80.4	187.6	268
	20	20.1	46.9	67
	100	4.02	9.38	13.4
0.45	1	603	737	1340
	5	120.6	147.4	268
	20	30.15	36.85	67
	100	6.03	7.37	13.4

DSD of produced emulsions was analyzed using Malvern Mastersizer 3000 (MS). In MS, the refractive index for RO and water was set to 1.466 and 1.33, respectively, for the lasers [red laser (632.8 nm) and blue laser (470 nm)]. Water was used as the dispersant medium at room temperature (20 °C). Thereafter, the emulsion was added into the dispersant tank where it was mixed with water and pumped through the flow cell where DSD was analyzed using the lasers. The obscuration level and constant stirring were maintained at 5–10% and 2500 rpm, respectively, as per the previous study by Upadhyay et al.^16^ The experiments with RO in water were performed three times to quantify error bars. The error bars on measured DSD and Sauter mean diameter (*d*_32_) values are included wherever possible. The potential sources of error in the experimental process may arise from errors in measuring flow rates, pressure drops, or droplet size distribution (DSD) values. The flow rates in the system were measured using a digital flow meter (sourced from Krohne Model: AF-E400) and the measurement accuracy was ±5%. The pressure drop was measured using a pressure gauge (Digitron, 2023P) with a pressure range of 0–300 kPA with an accuracy of ±5%. These instrument accuracy and errors in measurements will be reflected in DSD and *d*_32_. Each set of experiments for all devices was conducted three times to ensure reproducibility, with the associated errors of DSD and *d*_32_ found to be within ±10%.

Before conducting experiments with VD in the established setup for continuous production of emulsions, control experiments were carried out to quantify DSD of emulsions generated in the absence of VD (by the shear occurring in the T junction and subsequent loop configuration). For such control experiments, the flow rate through the loop (*Q*) was maintained at 1.35 LPM. RO in water emulsions produced at different values of *Q*/*q*_net_ (1, 5, 20, and 100) were characterized using a Mastersizer. Sample results are shown in Figure S2 (of the SI). It can be seen that in the absence of VD, the resulting emulsion is much coarser with an order of magnitude larger values of Sauter mean diameter (∼50 μm) than in the presence of VD. After establishing the key role of VD in producing fine emulsions, systematic experiments for quantifying the influence of *Q*/*q*_net_ and oil volume fraction on DSD were carried out.

### 2.2 Calibration of Turbidity Sensor

The results of Upadhyay et al.^16^ have shown that turbidity of emulsions [measured using off-line turbidity meter in terms of NTU (Nephelometric Turbidity unit)] is linearly proportional to oil volume fraction in the diluted sample (ε_O_). The turbidity (τ) and ε_O_ can then be related to DSD using the approach of Ranade and Ranade.^9^ In this approach, DSD of emulsions was represented by a sum of three log-normal distributions (with mean and variance for each distribution) and corresponding weightage fractions as eq 1
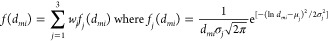
1where *d*_*mi*_ is a droplet diameter of *i*th bin, *w*_*j*_ are volume fractions of *i*th log-normal function, μ_*j*_ is the mean of the *i*th log-normal function, *f*_*j*_ (*d*_*mi*_)Δ*d*_*mi*_ is a volume fraction of oil droplets of *i*th population having diameters between *d*_*mi*_ and *d*_*mi*_+Δ*d*_*i*_, σ_*j*_^2^ is the variance of *i*th log-normal function, and σ_*j*_ is the standard deviation of the *i*th log-normal function. More details may be found in Upadhyay et al.^16^ or Section S7 in the Supporting Information. The turbidity can then be expressed as a function of discretized DSD and ε_O_ as eq 2
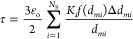
2where *N*_B_ is the number of bins into which DSD is divided, *K*_*i*_ is the scattering coefficient for droplets of size bin *i* with mean droplet diameter, *d*_*mi*_. At a higher ratio of droplet diameter to the wavelength of light, the value of the scattering coefficient is nearly constant and equal to 2. For lower ratios of droplet diameter to the wavelength of light, the value of scattering coefficient is a complex function of droplet diameter, wavelength, and the ratio of the refractive index of oil droplets to the refractive index of the continuous phase. If the value of *K*_*i*_ is approximated as 2α and eq 2 may be written as eq 3
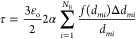
3

The Sauter mean diameter, *d*_32_, can be expressed in terms of *f*(*d*_*mi*_)Δ*d*_*mi*_ and *d*_*mi*_ (eq 4)
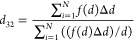
4

Combining eqs 3 and 4, we can relate turbidity with *d*_32_ as eq 5
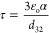
5

Typically, the Sauter mean diameter is related to the rate of change of turbidity with oil volume fraction as eq 6
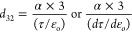
6The value of α was found to be 0.7.

The Sauter mean diameter of the emulsion was found to be inversely proportional to the rate of change of turbidity with oil volume fraction (eq 7)
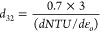
7

**Figure 2:**
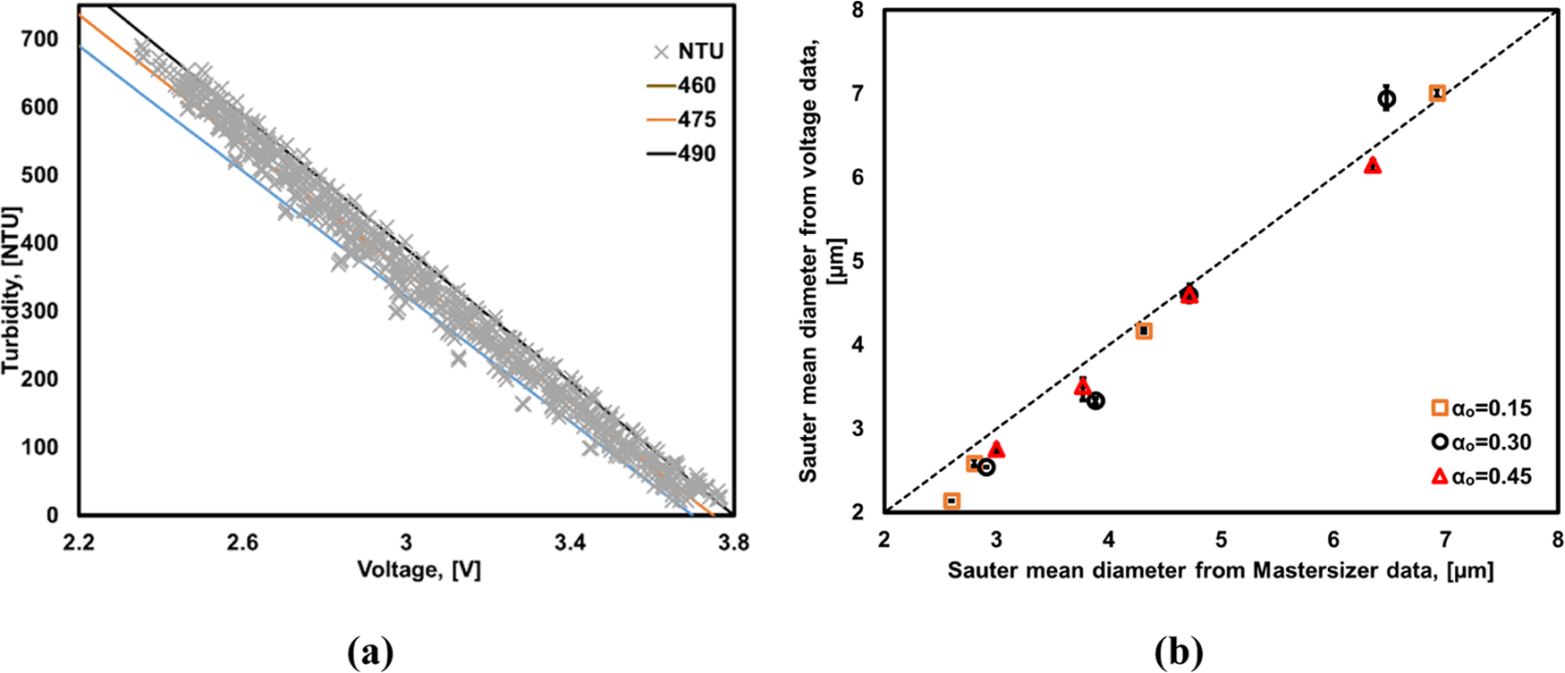
(a) Relationship between the measured voltage (*V*) and turbidity (NTU). (b) Parity plot of Sauter mean diameter obtained from eq 3 and Mastersizer.

Upadhyay et al.^16^ have used eq 7 to characterize the Sauter mean diameter of dense emulsions. Later, Ranade and Ranade^9^ used the values of turbidity and corresponding oil volume fraction for estimating the full DSD of emulsions using the ANN-based soft sensor. Leveraging these studies for interpreting data acquired from at-line turbidity meter, which provides output in terms of voltage between 0 and 5 V, needs to be related to turbidity measured in NTU. For this purpose, emulsions generated with oil volume fractions of 0.15, 0.30, and 0.45 with four different values of *Q*/*q*_net_ were diluted and turbidity was measured in terms of NTU and voltage at different values of oil volume fractions in the measured samples (see Section S3 in the Supporting Information for mode details on calibration experimental setup, procedure, and results). Based on the previous work of Upadhyay et al.,^16^ the suitable range of oil volume fraction (ε_O_) in the diluted stream was selected as 0.0002–0.0007. It should be noted that the at-line sensor exhibits the highest voltage at zero oil fraction in the measurement sample, and the value of voltage decreases with an increase in oil volume fraction in the sample. The measured values for NTU and voltage of emulsions are shown in Figure 2a. The relationship may be represented as eq 8
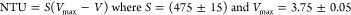
8The average values of slope, *S* (475) and maximum voltage, *V*_max_ (3.75) were used for further analysis. The influence of uncertainty in the values of *S* on the predicted DSD is discussed in Section 3.1.

Using the voltage data acquired for the emulsions produced in a continuous way and using eqs 7 and 8, the Sauter mean diameter may be related to voltage as shown in eq 9
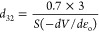
9

The comparison of the Sauter mean diameters estimated from the voltage data using eq 9 with those obtained from Mastersizer is shown in Figure 2b. Encouraged by the good agreement seen between the Sauter mean diameters obtained from Mastersizer and a voltage-based turbidity sensor, methodology for at-line estimation of DSD was developed as discussed in the following.

It should be noted that the calibration method linking turbidity (NTU) to voltage will depend on the refractive indices of dispersed oils. Therefore, recalibration is required to ensure accurate measurements across different oil types. However, the methodology presented here is generic and can be used for any emulsion containing different oils.

### 2.3 Development of a Methodology for At-Line Characterization of DSD

As mentioned earlier, the turbidity sensor used in this study gives the highest voltage (3.75 ± 0.05 V) with clear liquid, which gradually decreases as the oil volume fraction in the sample increases. The variation of voltage with oil volume fraction in the sample was found to be linear up to an oil volume fraction of 0.001. Considering that the emulsions being produced in continuous mode have much higher oil volume fractions (0.15–0.45) than this, it was essential to dilute the emulsions before measurements. A special arrangement of drawing a side-stream from the product outlet stream combined with continuous dilution was made as shown in Figure 1. The suitable range of oil volume fraction (ε_O_) in the diluted stream was selected (0.0002–0.0007) based on the previous work of Upadhyay et al.^16^ The following equation was used to select appropriate values of *q*_d_ and *q*_e_ (eq 10)
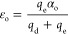
10

The diluent flow rates used in the present study were calculated using eq 10, which are listed in Table 2. *q*_dmax_ and *q*_dmin_ are maximum and minimum diluent flow rates, respectively.

**Table 2:** Dilution Flow Rates Used for At-Line Characterization of DSD

α_o_	10^4^ × ε_o_ (min)	10^4^ × ε_o_ (max)	*q*_e_ [mL/min]	*q*_dmin_ [mL/min]	*q*_dmax_ [mL/min]
0.15	2	7	2	428	1500
0.30	2	7	2	856	3000
0.45	2	7	1	642	2250

**Figure 3:**
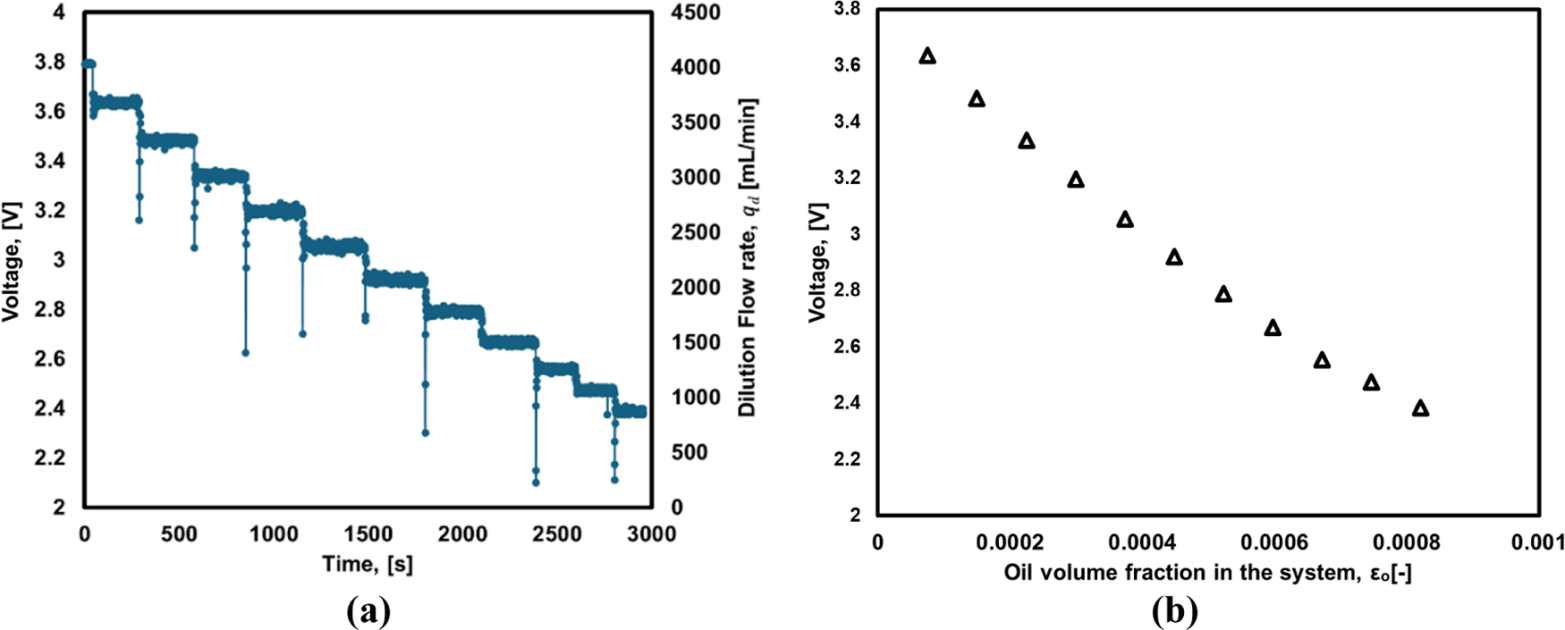
(a) Voltage and flow rate of the diluent stream (DI water) as a function of time. (b) Voltage as a function of oil volume fraction (ε_o_). Data are for 15% RO in water.

For at-line characterization, the emulsion is diluted by adjusting the flow rate of diluent, and the mixture is thoroughly mixed in a continuous stirred tank reactor (CSTR). The well-mixed diluted emulsion from this tank is then pumped through a turbidity sensor, where voltage values corresponding to the oil volume fraction were recorded. The diluent flow rates were changed manually by changing RPM of the peristaltic pump used for the diluent stream. During this process, the flow rates were varied from *q*_*dmax*_ to *q*_*dmin*_, and corresponding voltage readings were recorded over time. A sample of acquired voltage data with time where the flow rate of the diluent stream was varied in steps is shown in Figure 3a. These data are for *Q*/*q*_net_ = 100 and 15% oil in water emulsion. The oil volume fraction in a sample flowing through the at-line turbidity sensor (ε_O_) is shown in Figure 3b, which was calculated using eq 10.

Once the voltage data is acquired for the desired range of oil volume fraction, eq 2 was used to obtain turbidity in NTU. Ranade and Ranade^9^ developed artificial neural network model for relating turbidity, τ, and oil volume fraction in sample, ε_*O*_ with discretized DSD represented by a sum of three log-normal distributions (with mean and variance for each distribution) and corresponding weightage fractions as eq 1. Equation 1 contains nine parameters: −μ_*j*_, σ_*j*_, and *w*_*j*_ with *j* = 1, 2, and 3. The sum of weight fractions will always be one. Since the sum of volume fractions of three droplet populations is one, one parameter may be eliminated as eq 11
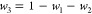
11

Thus, the full DSD of emulsions may be obtained with eqs 1 and 11 along with the eight parameters: −μ_*j*_ and σ_*j*_ with *j* = 1, 2, and 3 and *w*_*j*_ with *j* = 1 and 2. The ANN tool developed by Ranade and Ranade^9^ provides these eight parameters of DSD from the pair of turbidity (NTU) and oil volume fraction. In this study, we used the voltage acquired from the at-line sensor (after dilution as shown in Figure 1), eqs 8 and 10 along with ANN developed by Ranade and Ranade^9^ for estimating DSD. These results and their comparison with the experimental data are discussed in the following.

## 3 Results and Discussion

### 3.1 Comparison of Experimental and Predicted DSD

**Figure 4:**
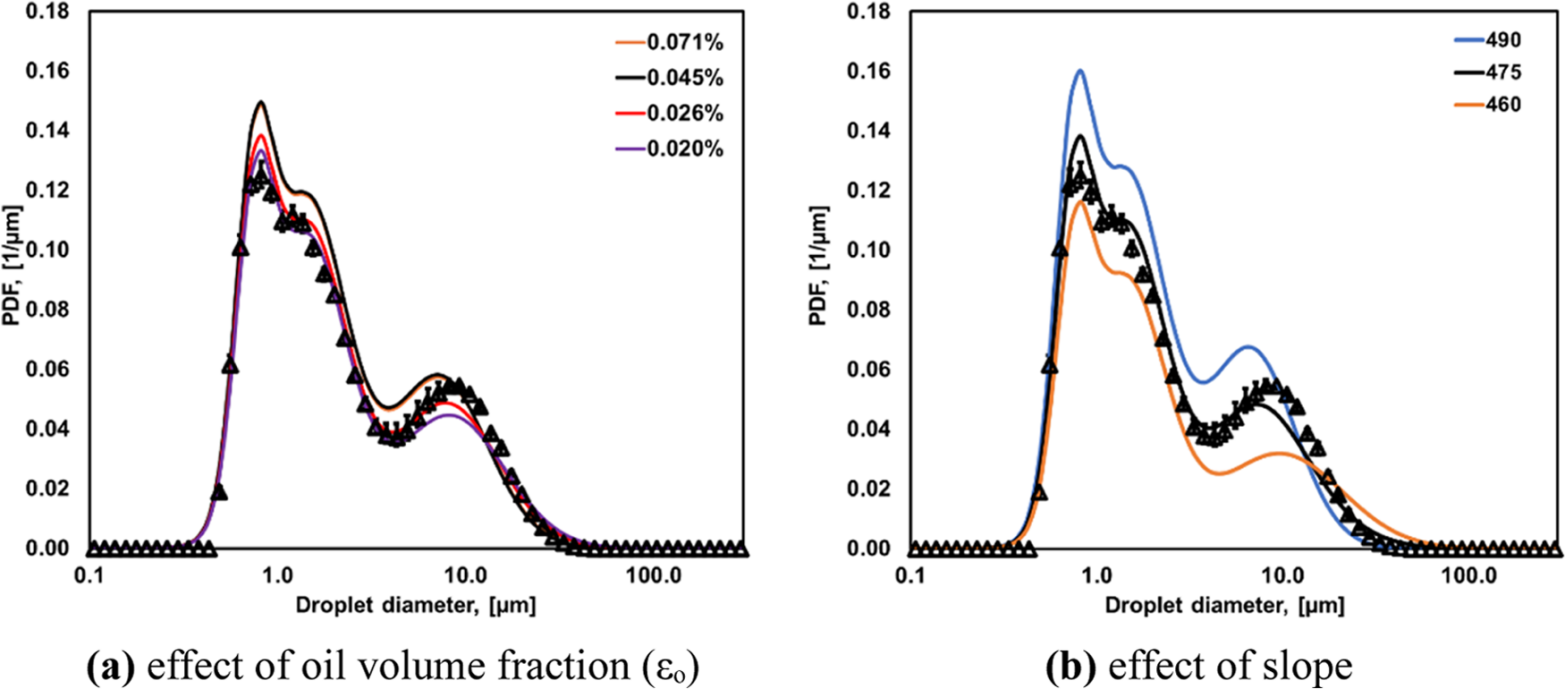
Comparison of DSDs predicted using ANN with those measured with Mastersizer. Sample case of emulsion with α_o_ = 0.30 for *Q*/*q*_net_ = 20. (a) Influence of oil volume fraction in the stream flowing through turbidity meter (ε_o_) with *S* = 475. (b) Influence of parameter *S* of eq 2 = 0.00045. Experimental DSD data are represented by symbols, and ANN-predicted DSDs are represented by lines.

The DSD data was collected for 12 different emulsions produced in a continuous mode, each characterized by a specific oil volume fraction and *Q*/*q*_net_. For each of these emulsions, at-line measurements were carried out using four different diluent flow rates to get the percentage of oil volume in the measurement samples as 0.020, 0.03, 0.045, and 0.071%. The voltage measured at these oil volume fractions was converted to turbidity (NTU) using eq 8 and the turbidity and oil volume fraction were used with the trained ANN developed by Ranade and Ranade^9^ to estimate the corresponding DSDs. The comparison of DSD estimated from the ANN tool using the mean value of *S* (475) and the experimental data for the four different values of ε_*O*_ is shown in Figure 4a. It can be seen that DSD estimated from the at-line measurements of voltage and ANN tool with *S* = 475 agree very well with DSD measured by Mastersizer. It can also be estimated that DSD is not very sensitive to the value of ε_O_, and therefore any value of ε_O_ within the selected range may be used. At-line measurement of voltage with just one diluent flow rate may thus provide an adequately good estimate of DSDs. All subsequent estimations of DSDs were then conducted using voltage measurements at just one dilution rate (with corresponding range of ε_O_ as 0.0002–0.0007).

**Figure 5:**
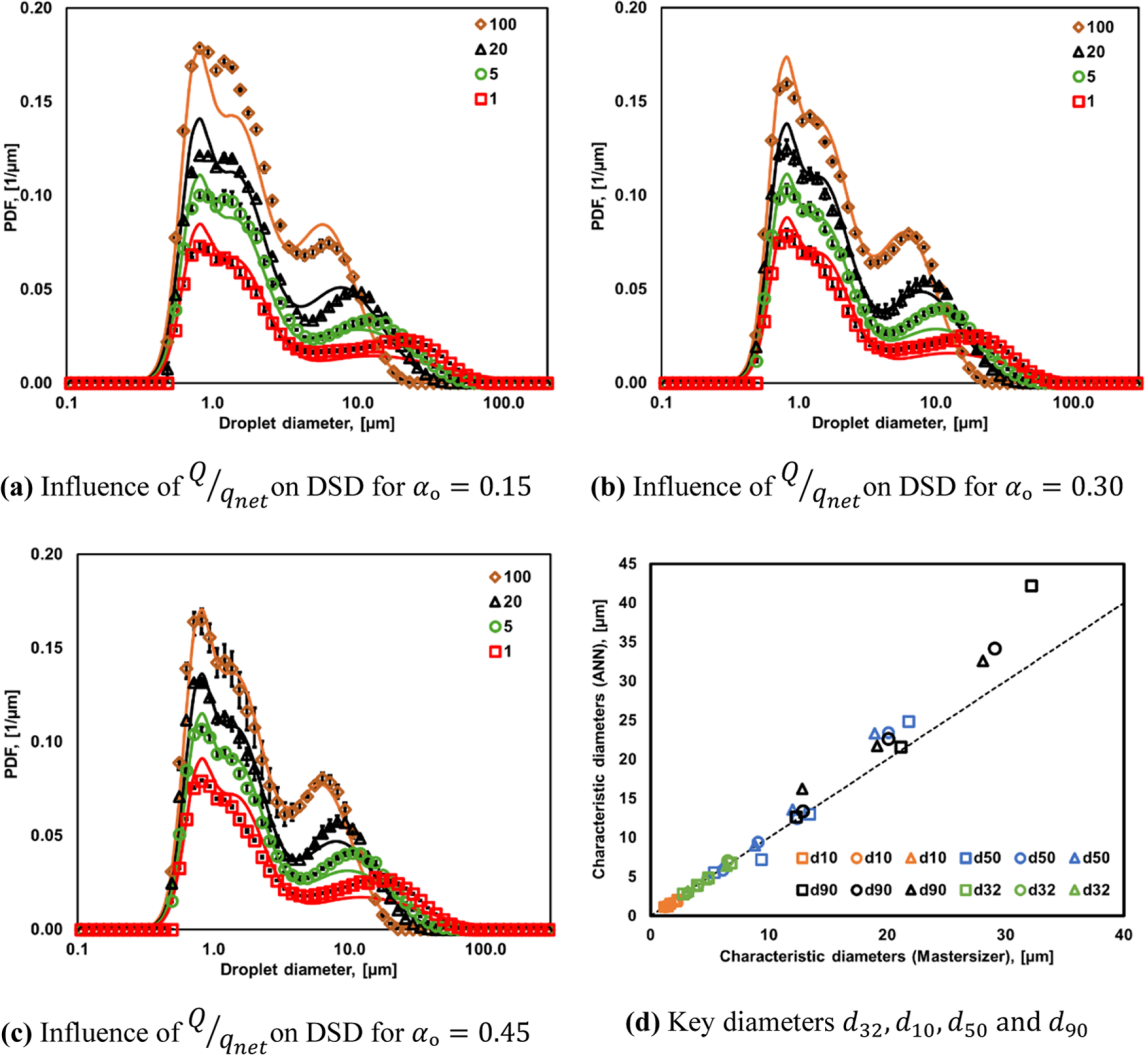
Comparison of the predicted DSD (represented by lines) with the experimentally measured DSD (represented by symbols).

The comparison of DSDs estimated using an at-line turbidity sensor and ANN with the Mastersizer results for all of the emulsions considered in this study is shown in Figure 5a–c. It can be seen that DSDs estimated from at-line measurements of voltage and trained ANN agree with the experimental data quite well. Full DSDs estimated from these measurements were used for estimating key characteristic diameters such as *d*_32_, *d*_10_, *d*_50_, *and d*_90_. The comparison of characteristic diameters obtained from Mastersizer and those calculated from estimated DSDs is shown in Figure 5d. It can be seen that barring the overprediction of *d*_90_ at *Q*/*q*_net_ = 1, all of the other characteristic diameters were predicted very well. The deviation in *d*_90_ values of *Q*/*q*_net_ = 1 is because of a lack of agreement with the tail toward larger diameters. The deviation in *d*_90_ or tail toward larger diameters is reflected in Span and PDI of predicted DSD for *Q*/*q*_net_ = 1. The comparison of the Span and PDI of experimental and predicted (ANN) DSD is given in Section S6 in the Supporting Information. The comparison of the Sauter mean diameter of the Mastersizer and those predicted from DSD obtained with ANN show very good agreement. The Sauter mean diameter (*d*_32_) arises from its focus on the overall surface-weighted average, making it less sensitive to errors in the distribution tail toward larger diameters. However, metrics such as *d*_90_, Span, and PDI are influenced by such a tail, leading to greater discrepancies when large particles are overpredicted. The values of Span and PDI obtained from experimentally measured DSD as well as DSD predicted by ANN are listed in Table S5 of the Supporting Information. Despite some of these discrepancies, the *R*^2^ value for all of the experimental and estimated characteristic diameters is ∼0.96, which may be considered as adequate. The *R*^2^ values calculated from the comparison of experimental and predicted (ANN) DSD are listed in Table S2 of the Supporting Information. ANN developed and validated using the experiments conducted in a batch mode (Ranade and Ranade^9^) was able to predict DSD of emulsions generated in a continuous mode. It will therefore be instructive to compare the comparison of DSD of emulsions generated in batch and continuous modes. These results are discussed in the following section.

### 3.2 Performance of Continuous Production of Emulsions

**Figure 6:**
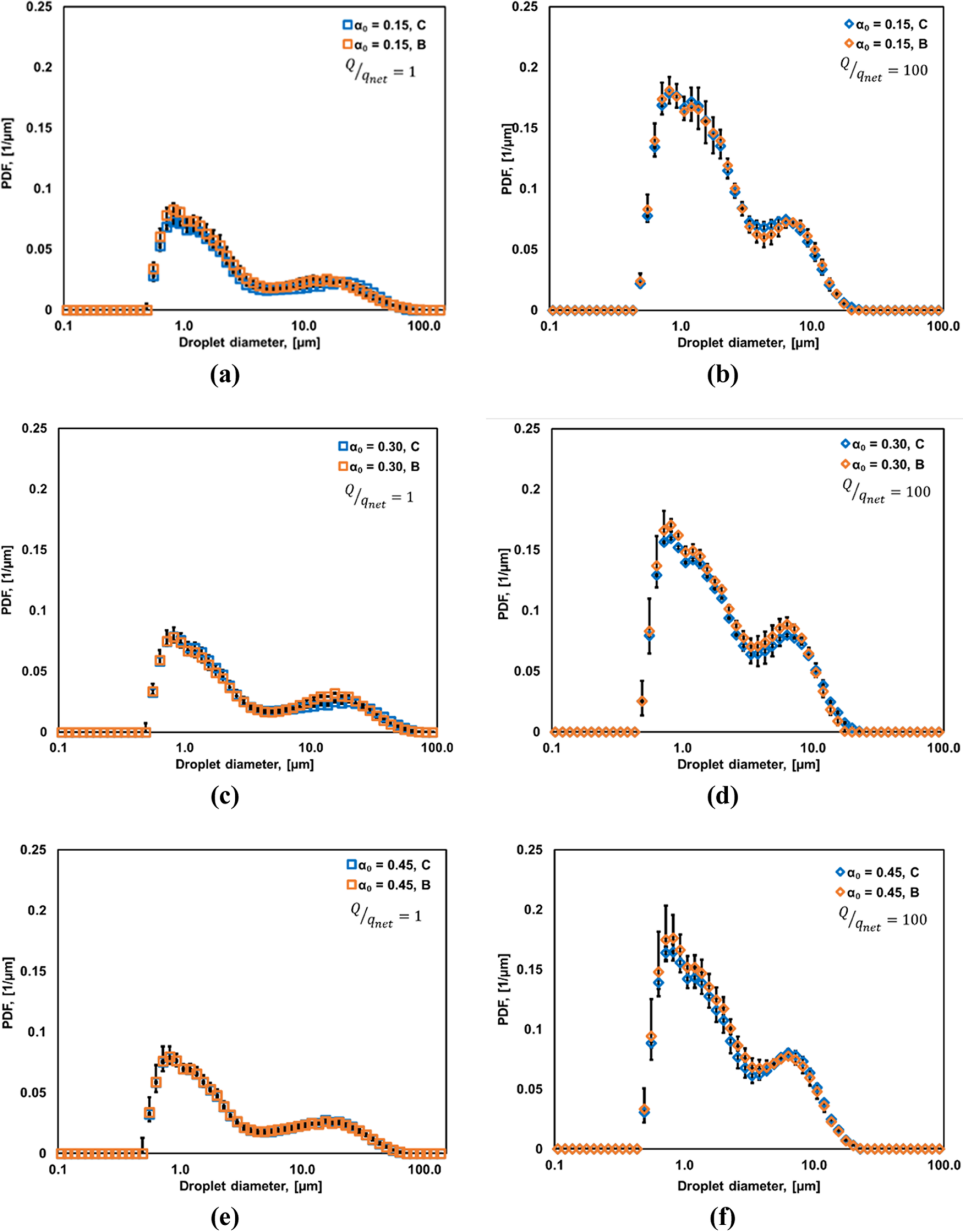
DSD comparison of batch experiments (B) and continuous experiments (C) using the same vortex-diode, for α_O_ = 0.15 (a) & (b), α_O_ = 0.30 (c) & (d), and α_O_ = 0.45 (e) & (f) with *Q*/*q*_net_ = 1 and 100.

Before discussing the performance of continuous production of emulsions, the comparison of DSD and *d*_32_ of the emulsions produced in batch and continuous modes is presented. For this, the same vortex-based cavitation device used for the continuous generation of emulsions was used for generating emulsions in a batch mode of operation (using a setup similar to that described in our earlier work, Upadhyay et al.^16^). For establishing a uniform basis for comparison of DSDs obtained with batch and continuous modes of operation, we selected the energy consumption per unit weight of emulsions as a basis. The energy consumption per unit weight of emulsion in batch mode (*E*_B_) and continuous mode (*E*_C_) was calculated using eqs 12 and 13, respectively
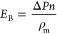
12
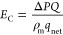
13

where Δ*P* is the pressure drop across the device, *Q* is the flow rate through the device, ρ_*m*_ is the mixture density, *n* is the number of passes (for the batch mode), and *q*_net_ is the net flow rate of emulsion (for the continuous mode). By comparing eqs 12 and 13, it can be seen that at equal energy consumption per unit weight of emulsion, *n* = *Q*/*q*_net_. By appropriately selecting values of *n* and *Q*/*q*_net_, it was possible to ensure the same energy consumption per unit mass of produced emulsion in the batch and continuous modes (see Table S8 of the Supporting Information). The comparison of DSD obtained with batch and continuous modes of operation for *Q*/*q*_net_ = 1 and 100 of all oil volume fractions (α_O_ = 0.15, 0.30, and 0.45) is shown in Figure 6a–c.

**Figure 7:**
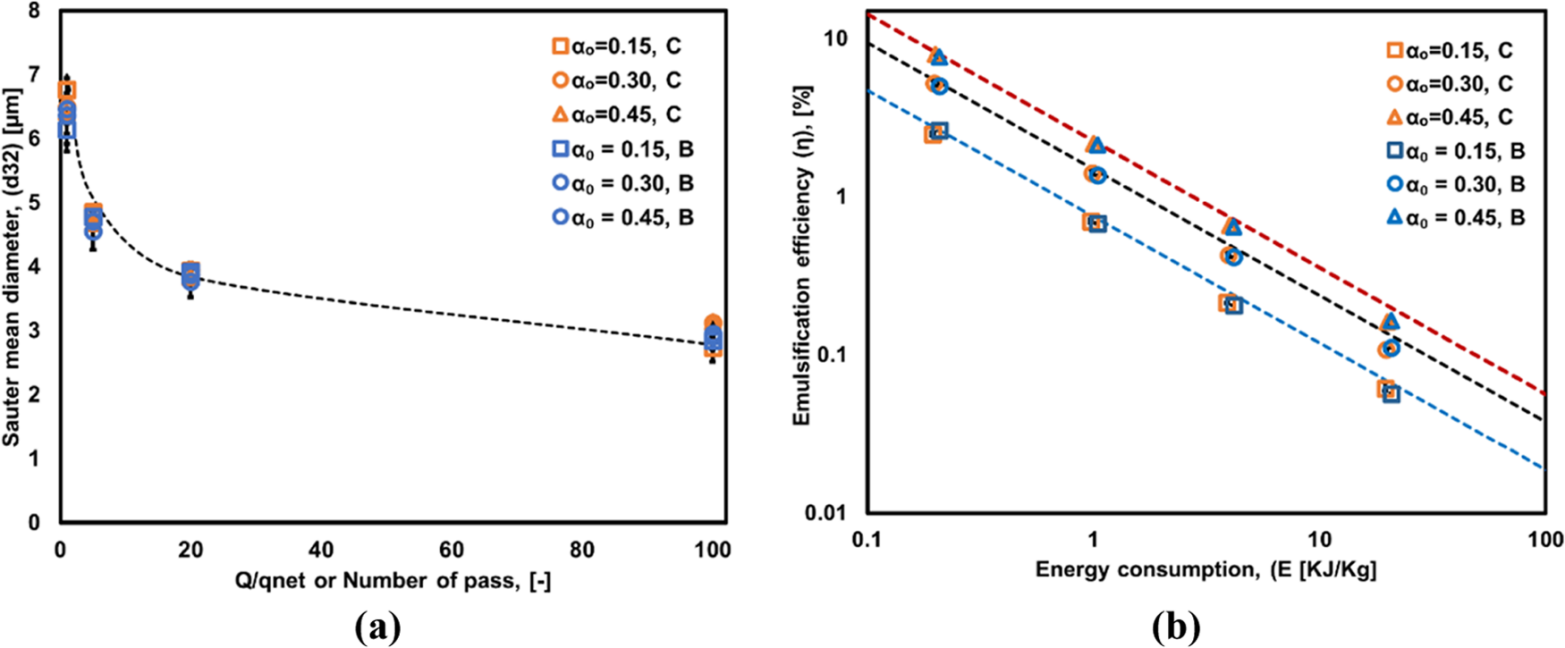
Comparison of (a) Sauter mean diameter (*d*_32_) and (b) emulsification efficiency (η) of emulsions produced in continuous (C) and batch (B) modes. The symbols represent the experimental data, and dashed lines indicate eq 14 for *d*_32_ and eq 15 for η.

It can be seen that emulsions produced in a batch or continuous state exhibit almost the same DSD if operated with the same energy consumption per unit mass. This is also reflected in the comparison of the Sauter mean diameters and emulsification efficiency obtained with batch and continuous modes of operation as shown in Figure 7. The Sauter mean diameters of both the production modes may be represented (shown as dashed lines in Figure 7) as in eq 14
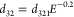
14where *d*_321_ is the Sauter mean diameter with *E* = 1 kJ/kg. The values of *d*_321_ for both modes of operation were found to be 6.5 μm. Note that this value of *d*_321_ is about 20% larger than that reported by Upadhyay et al.^16^ (*d*_321_ = 5.5 μm). This difference is because of the different flow characteristics of the devices used in the present study with that used by Upadhyay et al.^16^ The Euler number  of VD used in the present study was lower (35) than that used in the work of Upadhyay et al.^16^ (42). VD with a higher (20%) Euler number was found to lead to smaller droplets (about 20%). The comparison of DSD and *d*_32_ obtained in the present study (batch mode) and the batch data from Upadhyay et al.^16^ is shown in Figure S7 of the Supporting Information.

The emulsification efficiency for the modes of operation based on DSDs obtained from VD used in the present study can be correlated as shown in eq 15
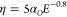
15

It can be seen that eq 15 represents the experimental data quite well. The emulsification efficiency thus was found to be proportional to oil volume fraction over the range considered in the present study (up to 0.45 volume fraction of oil). As energy consumption increases, emulsification efficiency decreases as per eq 15.

It was shown that the vortex-based cavitation device operated in a flow loop can be used for producing dense oil in water emulsions in continuous mode. The developed methodology of using an at-line turbidity meter and ANN-based soft sensor of Ranade and Ranade^9^ was able to estimate full DSD and thereby key characteristic diameters quite well. The turbidity model and the soft sensor developed based on that are applicable for any operating pressure of the considered vortex-based cavitation device or any oil volume fraction. If a different emulsification device than VD is used or a different oil phase is used, it will be necessary to recalibrate the ANN-based soft sensor as well as the voltage sensor (with respect to NTU). The methodology presented in this study and that presented by earlier work of Ranade and Ranade^9^ may be used for such recalibration. The overall approach is quite general and applicable for any emulsification device and emulsion system. The presented results and approach of estimating DSD using an inexpensive at-line turbidity sensor will provide a sound basis for further research on controlling DSD of emulsions in decentralized, continuous, and on-demand production of tailored emulsions.

## 4 Conclusions

Vortex-based cavitation device operated in a flow loop was developed and used for producing dense oil in water emulsions (with oil volume fraction up to 0.45) in a continuous mode. An approach for estimating at-line DSD of such dense emulsions was developed using an inexpensive voltage-based turbidity sensor and a previously developed ANN-based soft sensor. Application of this approach was successfully demonstrated for emulsions of rapeseed oil in water over a wide range of oil volume fractions and operating conditions. The key conclusions of this study are the following:

- The emulsions produced using a vortex-based hydrodynamic cavitation device in a continuous model exhibited bimodal DSD similar to those observed for emulsions produced in a batch mode.
- The voltage acquired from the turbidity sensor used in this study exhibited a linear relationship with the oil volume fraction in the measured emulsion. The acquired voltage was found to be linearly related (eq 8) to turbidity measured using an off-line turbidity meter (in NTU).
- The ANN-based soft sensor developed by Ranade and Ranade^9^ was able to estimate full DSD of emulsions using at-line measurements conducted using the turbidity meter operated with the appropriate diluent stream. The predicted DSD and key characteristic diameters such as *d*_32_, *d*_10_, *d*_50_, and *d*_90_ showed good agreement with the data obtained from Mastersizer.
- DSDs of emulsions produced in a continuous mode agree very well with DSDs of emulsions produced in a batch mode using the same emulsification device. The Sauter mean diameter was found to be independent of oil volume fraction (α_O_) and inversely proportional to energy consumption (as *E*^–0.2^). The emulsification efficiency was found to be directly proportional to the oil volume fraction (α_O_) and proportional to the energy consumption as *E*^–0.8^.

The developed approach of at-line characterization of DSD and presented results for continuous production of dense oil in water emulsions will be useful for developing decentralized production of tailored emulsions.

## Supporting Information Available

Photograph of the continuous emulsion production with at-line characterization, experiments without hydrodynamic device (vortex-diode), experimental setup and procedure for calibrating turbidity sensor, comparison of experimental and predicted DSDs in terms of *R*^2^ values, comparison of batch data from Upadhayay et al.^16^ and present continuous data, continuous Span from experimental and predicted data, DSD representation using log-normal function, and energy consumption data for batch and continuous modes of emulsion production (PDF)

## Nomenclature

*d*_32_: Sauter mean diameter (μm)
*d*_*i*_: diameter of the *i*th droplet (μm)
*d*_90_: droplet diameter at 90 vol % in the DSD curve (μm)
*d*_10_: droplet diameter at 10 vol % in the DSD curve (μm)
*d*_50_: median of volume distribution; 50 vol % in the DSD curve (μm)
*d*_*T*_: throat diameter (mm)
*E*: energy consumption per unit mass of emulsion (J/kg)
*Eu*: Euler number (−)
*K*: light scattering coefficient
*N*_*i*_: concentration of particle
*n*: number of pass (−)
Δ*P*: inlet pressure drop (kPa)
*Q*: volumetric flow rate in recirculation loop (m^3^/s)
*q*_oil_: volumetric flow rate of oil (m^3^/s)
*q*_water_: volumetric flow rate of water (m^3^/s)
*q*_e_: volumetric flow rate of the emulsion (m^3^/s)
*q*_d_: volumetric flow rate of the dilution stream (m^3^/s)
*Re*: Reynolds number (−)
S: slope (−)
*T*: temperature (°C)
*t*: time (s)
*V*: voltage
*V*_T_: throat velocity (m/s)

## Greek Letters

α_o_: oil volume fraction (−)
ρ_O_: density of rapeseed oil (kg/m^3^)
ρ_w_: density of water (kg/m^3^)
μ_O_: viscosity of rapeseed oil (mPa·s)
μ_w_: viscosity of water (mPa·s)
σ: interfacial tension (N/m)
η: energy efficiency for droplet breakage (%)
τ: turbidity (1/m)
ε_o_: oil volume fraction in the dilution stream (−)

## Acronyms

ANN: artificial neutral networks
CSTR: continuously stirred tank reactor
DSD: droplet size distribution
HC: hydrodynamic cavitation
NTU: nephelometric turbidity units
RO: rapeseed oil
VD: vortex diode
PDF: probability density function
