# Supplementary material for: At-Line Characterization of Droplet Size Distributions Using a Simple, Voltage-Based Sensor for Continuous Production of Dense Oil in Water Emulsions
Source: Ind Eng Chem Res. 2025 Feb 7;64(7):4105–15. doi: 10.1021/acs.iecr.4c03979 (PMC11843604; doi:10.1021/acs.iecr.4c03979)
Supplement: Supplementary file 1 — ie4c03979_si_001.pdf [file ie4c03979_si_001.pdf]

Supplementary Information

At-line characterisation of droplet size distributions using a simple, voltage-based sensor for continuous production of dense oil in water emulsions

Akshay Ravi, Amol V. Ganjare and Vivek V. Ranade*
Multiphase Reactors and Intensification Group
Bernal Institute, University of Limerick, Limerick V94T9PX, Ireland
*Email: vivek.ranade@ul.ie

S1: Photograph of the continuous emulsion production with at-line characterisation.

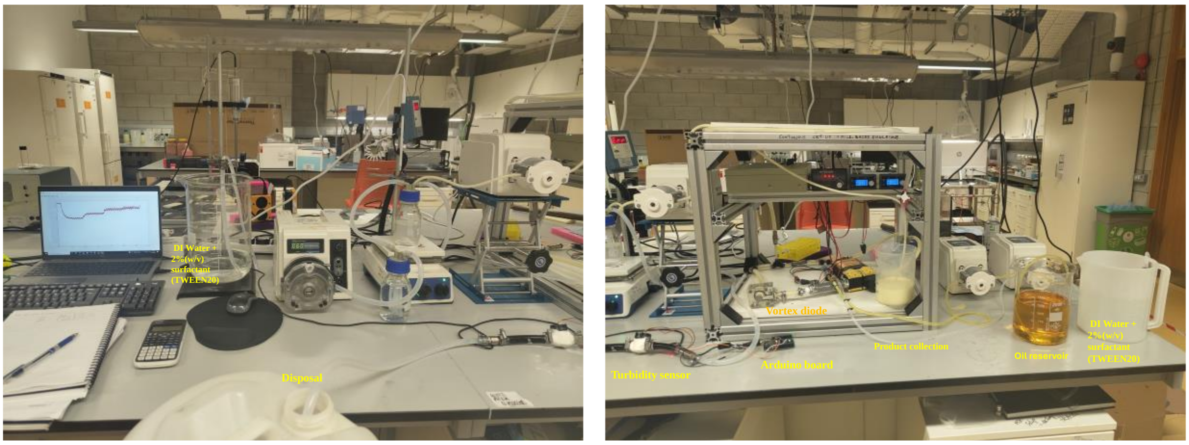

(a) **(b)**

Figure S1. Experimental setup **(a)** at-line turbidity-based soft sensor and (**b)** continuous emulsion production system.

The experiment geometry, materials, operation conditions, and information on the experiments conducted are given below Table S1.

Table S1: The experimental setup information, operating parameters, and materials

**Parameter**	**Value**
Length of tubing1 $[mm]$	200
Length of tubing2 $[mm]$	500
Diameter of tubing1 $[mm]$	7.9
Diameter of tubing2 $[mm]$	7.9
Volume in holding tank $[mL]$	60
The volume of tubing1 $[mL]$	9.8
The volume of tubing2 $[mL]$	24.5
The volume of water in the recirculation system, V $[mL]$	96.1
Throat diameter of HC device, d_T $[m]$	0.003
Rapeseed oil (RO) density, ρ_O $[kg/m^3]$	915
Rapeseed oil viscosity, μ_O $[mPa.S]$	62
Water density, ρ_W $[kg/m^3]$	997
Water viscosity, μ_W $[mPa.S]$	0.7972
Water-RO interfacial tension, σ $[mN/m]$	35
Temperature, T [°C]	~20
Pressure drop, ΔP [kPa]	200
Q/q_{net} parameter	1,5,20 &100

S2. Experiments without hydrodynamic device (vortex-diode)

We have conducted experiments without the vortex-diode to quantify the coarse emulsion produced before the vortex-diode. The recirculation circuit was maintained at same flow rate (Q=1.35LPM) as with vortex-diode then for different $Q/qnet$=1, 5, 20 and 100 emulsions were obtained and using Master-sizer with same settings the DSDs were obtained. Then below is comparison of same $Q/qnet$ for with vortex-diode and without vortex-diode. The DSD of coarse emulsions without vortex-diode are almost similar with Sauter mean diameter ~50μm.

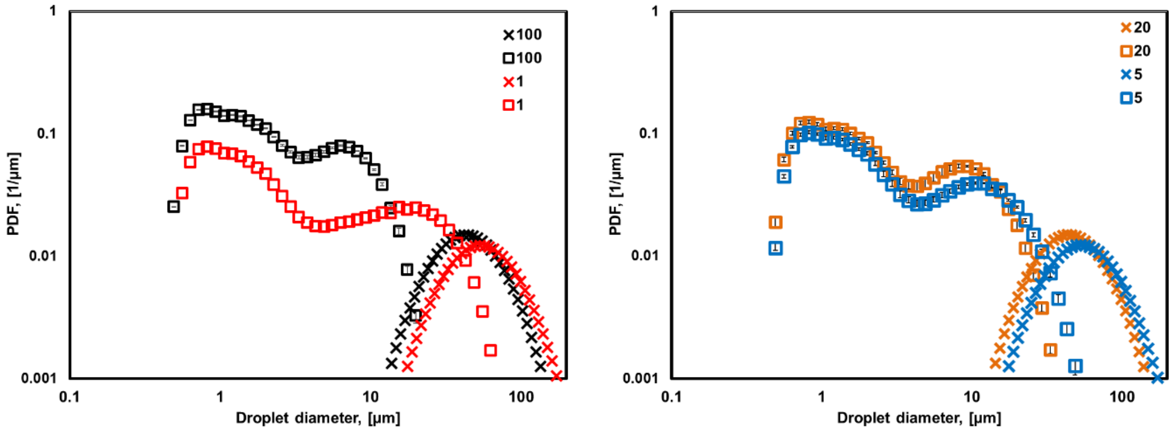

(a) (b)

Figure S2. The comparison of DSD of the emulsions produced at same Q/q_{net} with vortex-diode (square symbols) and without vortex-diode (cross symbols) for oil volume fraction (α_O) 0.3.

S3: Experimental setup and procedure for calibrating turbidity sensor.

In this section, we have given information on the design of the experimental setup to calibrate the turbidity sensor (DF Robot) using a commercial turbidimeter (VELP Scientifica, Italy). Figure S2 (a) & (b) shows the schematic and actual picture of the experimental setup. The emulsion in the beaker was stirred continuously using a magnetic stirrer and circulated through the turbidity sensor using a peristaltic pump (Longer Model WT600J). The turbidity sensor voltage values were acquired using an Arduino Board for different dilutions or oil volume fractions (ε_o) and emulsions. The turbidity sensor gives output in terms of voltage between 0 to 5V, and these voltage values need to be calibrated with commercial turbidimeter data in terms of NTU (nephelometric turbidity units).

The experimental procedure is explained as follows: Initially, the 1000 mL Deionised water with 2% (w/v) surfactant TWEEN20 (sourced from MP Biomedicals, France) was stirred continuously in the beaker using a magnetic stirrer. The flow was pumped through the turbidity sensor and back to the beaker using a peristaltic pump. The pump was operated at the flow rate of 1800mL/min. The emulsion of oil volume fractions α_o=0.15 and 0.30 with samples collected for four numbers of passes (n=1,5,20 and 100) through the vortex-diode (Hydrodynamic Cavitation device) were selected for the calibration. These emulsions are selected because oil volume fractions are below the critical volume fraction, α_o=0.35, where d_{32} is independent of the oil volume fraction.

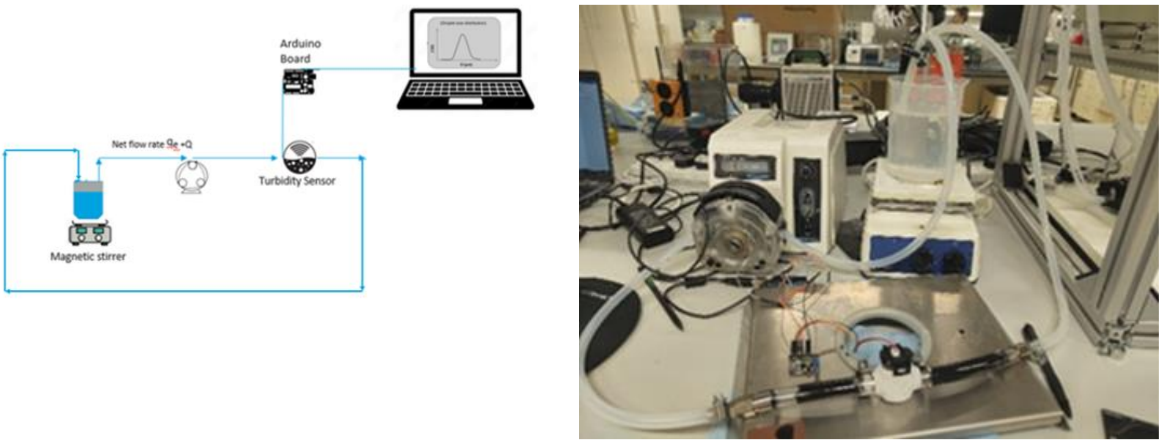

(a) **(b)**

Figure S3. Calibration experimental details **(a)** schematic diagram and **(b)** actual photograph.

An initial study was conducted to understand the effect of flow rate and surfactant mixed with DI water on voltage from the Turbidity sensor. The flow rate of DI water was increased stepwise from 500mL/min to 3200mL/min, and then surfactant was mixed in DI water, and respective voltage readings were acquired. The data was acquired using an Arduino Board, and the output file was written using MATLAB.

After these initial studies, emulsions were added stepwise into the 1L DI water system with a surfactant, and the flow rate was set at 1800mL/min. The volume of the emulsion sample introduced was 0.5 mL for emulsion with α_o=0.15 and 0.25 mL for emulsion with α_o=0.30. The continuous voltage data acquisition and output file was written using Arduino Board and MATLAB for 4-5 minutes with a 1-second interval. After 3 minutes of addition of each sample emulsion into the system, 10 mL solution is taken from the system using a micropipette, and NTU is measured by a commercial Turbidity meter. Later, the solution is poured back into the system. The continuous voltage and NTU are measured or acquired for each stepwise addition of the selected emulsion until the NTU value reaches around 600.

S3.1. Comparison of slope from NTU and voltage.

The slope obtained from NTU versus oil volume fraction (ε_O) and slope from voltage values converted to NTU (NTUv) using the calibration equation are compared.

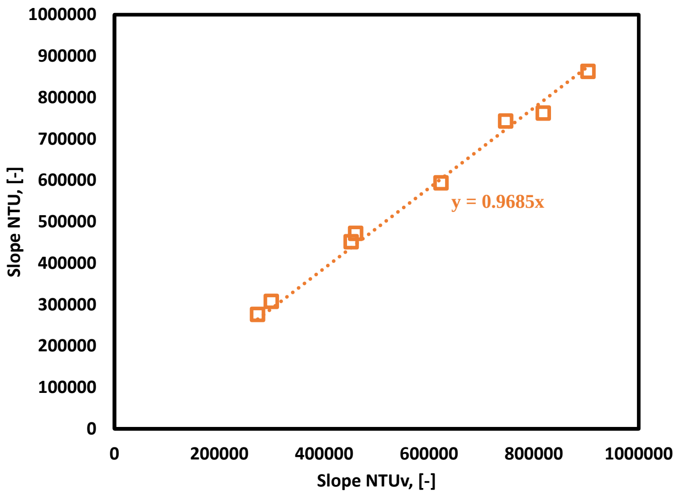

Figure S4. Combined slope values based on NTU and Voltage for emulsions α_o=0.15 and α_o=0.30

The slope and d_{32} data were compared with a number of passes of the sample emulsions to show the calibration equation developed was in good agreement with previous studies' estimations.

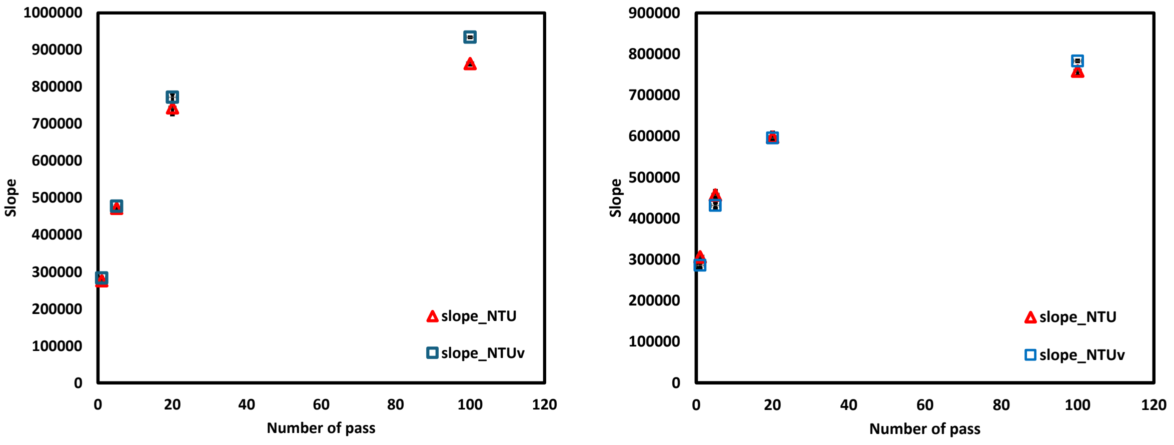

(a) (b)

Figure S5. Slopes from NTU and Voltage compared with emulsion sample obtained at a respective number of passes for **(a)**α_o=0.15 and **(b)** α_o=0.30

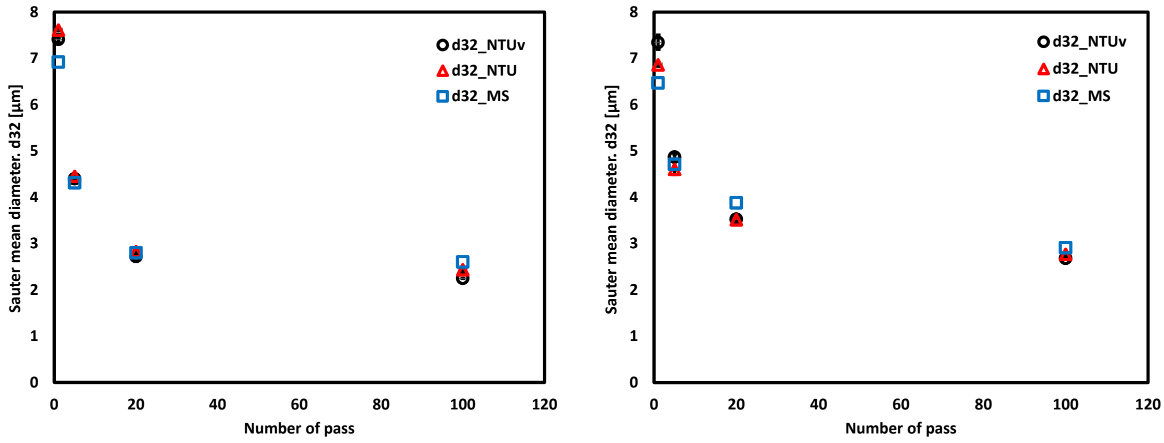

(a) (b)

Figure S6. Sauter mean diameter from NTU, Voltage, and Master-sizer (MS) compared with emulsion sample obtained at a respective number of passes for **(a)** α_o=0.15 and **(b)** α_o=0.30

S4. Comparison of experimental and predicted DSD in terms of R² values.

Table S2. Comparison of predicted with experimental DSD for $\alpha_o = 0.15$

$\frac{Q}{q_{net}}$	ε_o	R^2
100	0.000356	0.99
	0.000227	0.99
	0.000131	0.99
	0.0000999	0.98
20	0.000356	0.99
	0.000227	0.99

	0.000131	0.98
	0.0000999	0.98
5	0.00071	0.99
	0.00045	0.99
	0.00026	0.98
	0.000199	0.98
1	0.00071	0.97
	0.00045	0.97
	0.00026	0.97
	0.000199	0.97

Table S3. Comparison of predicted with experimental DSD for $\alpha_o = 0.30$

$\frac{Q}{q_{net}}$	ε_o	R^2
100	0.00071	0.99
	0.00045	0.99
	0.00026	0.999
	0.000199	0.996
20	0.00071	0.99
	0.00045	0.99
	0.00026	0.99
	0.000199	0.98
5	0.00071	0.99
	0.00045	0.99
	0.00026	0.98
	0.000199	0.98
1	0.00071	0.97
	0.00045	0.97
	0.00026	0.96
	0.000199	0.96

Table S4. Comparison of predicted with experimental DSD for $\alpha_o = 0.45$

$\frac{Q}{q_{net}}$	ε_o	R^2
100	0.001069	0.98
	0.0000681	0.99
	0.000394	0.99
	0.0002998	0.99
20	0.001069	0.99
	0.0000681	0.99
	0.000394	0.99
	0.0002998	0.98
5	0.001069	0.99
	0.0000681	0.99
	0.000394	0.98
	0.0002998	0.98
1	0.001069	0.97
	0.0000681	0.97
	0.000394	0.97
	0.0002998	0.96

S5. Comparison of DSD and Sauter mean diameter (d_{32}) of batch and continuous mode

In this section, DSD and d_{32} of batch and continuous mode produced emulsions compared with previous published data by Upadhayay et al.[16] .This study or comparison was done to show the consistency of batch and continuous emulsions with different vortex-diodes. The Euler number of the device used in the work of Upadhayay et al.[16] was 42 as compared to 35 of the present work.

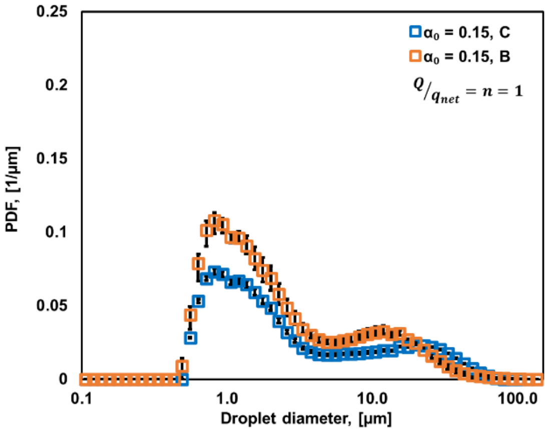

(a)

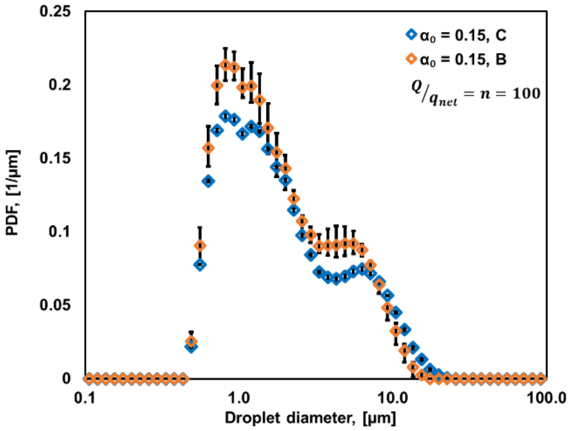

(b)

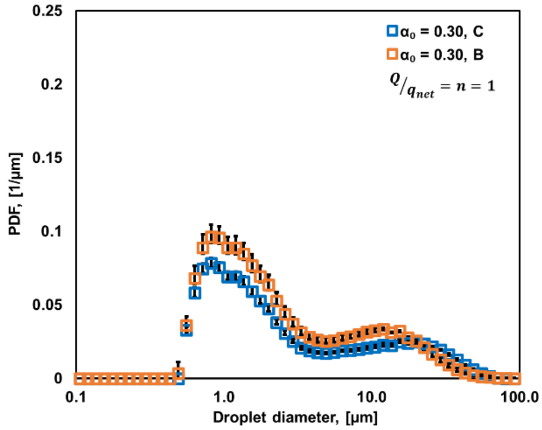

(c)

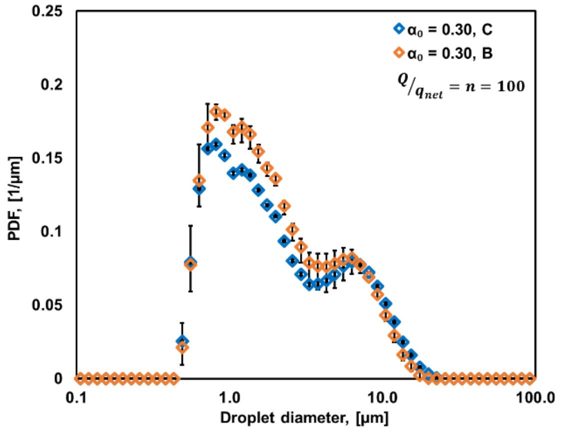

(d)

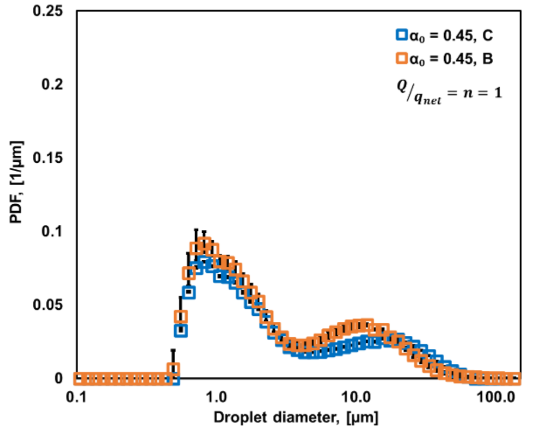

(e)

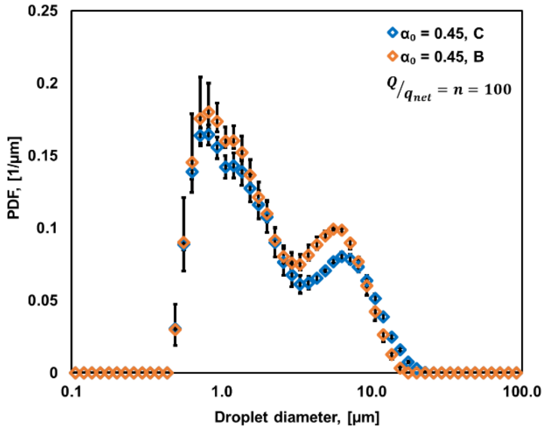

(f)

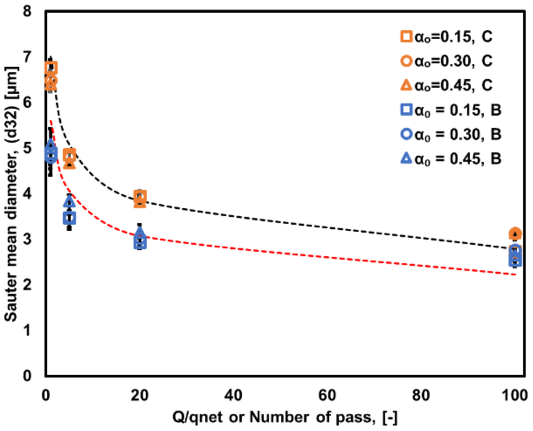

(g)

Figure S7. DSD comparison of batch experiments data (B) of Upadhayay et al.[16] and continuous experiments (C), for $\alpha_O = 0.15$ **(a)** & **(b)**, $\alpha_O = 0.30$ **(c)** & **(d)** and $\alpha_O = 0.45$ **(e)** & **(d)** with $Q/q_{net} = 1$ and 100. **(g)** Sauter mean diameter (d_{32}) comparison.

S6. Comparison of Span and PDI from experimental and predicted data.

Table S5. Comparison of experimental values of Span and PDI with those obtained from ANN-based DSD

α_O	Q/q_{net}	Span (Experimental)	PDI (Experimental)	Span (ANN)	PDI (ANN)
0.15	1	1.99	2.43	3.47	7.43
	5	2.28	2.18	3.11	3.37
	20	2.11	1.63	2.81	1.85
	100	2.09	1.11	2.06	1.13
0.3	1	2.05	2.35	2.85	6.62
	5	2.22	1.95	2.62	3.34
	20	2.06	1.54	2.25	1.94
	100	1.91	1.11	2.07	1.19
0.45	1	2.05	2.24	3.34	5.99
	5	2.21	1.94	2.28	3.04
	20	2.02	1.52	2.25	2.01
	100	1.85	1.12	2.22	1.23

S7. DSD representation using log-normal function

The expanded version of Equation (1) of the manuscript is as follows:

$$f_1(d_{mi};\mu_1,\sigma_1) = \frac{1}{d_{mi}\sigma_1\sqrt{2\pi}}\exp\left(-\frac{(\ln(d_{mi})-\mu_1)^2}{2\sigma_1^2}\right) \quad \text{(S1)}$$

$$f_2(d_{mi};\mu_2,\sigma_2) = \frac{1}{d_{mi}\sigma_2\sqrt{2\pi}}\exp\left(-\frac{(\ln(d_{mi})-\mu_2)^2}{2\sigma_2^2}\right) \quad \text{(S2)}$$

$$f_3(d_{mi};\mu_3,\sigma_3) = \frac{1}{d_{mi}\sigma_3\sqrt{2\pi}}\exp\left(-\frac{(\ln(d_{mi})-\mu_3)^2}{2\sigma_3^2}\right) \quad \text{(S3)}$$

$$f(d_{mi}) = w_1 f_1(d_{mi};\mu_1,\sigma_1) + w_2 f_2(d_{mi};\mu_2,\sigma_2) + (1-(w_1 + w_2))\, f_3(d_{mi};\mu_3,\sigma_3) \quad \text{(S4)}$$

The size of droplets in a suspension can be estimated by measuring the turbidity of the suspension. Turbidity measures the attenuation of a beam of light traveling through the suspension caused by the scattering and absorption of light by the droplets. The amount of scattering and absorption depends on the size of the droplet and their concentration in the suspension. In standard spectrophotometer, light absorbed by droplets related to the droplet size. The transmitted light measured by a standard spectrophotometer which reports absorbance as[16]:

$$A = \frac{1}{l_{path}}\log\frac{I_{in}}{I} \quad \text{(S5)}$$

Where, I is transmitted light intensity and I_{in} is the incident light intensity. The log is of base 10. Spectrophotometer cuvettes light transmission path of 0.01 m and reports values of absorbance (A) for different wavelengths. The unit of A therefore is m^{-1}. Unlike the UV-Vis spectrophotometer, commercial turbidity meters measure turbidity in NTU by measuring scattered light at 90° to the direction of light beam. The effective turbidity may also be related to detected light intensity (I) as[16].

$$\tau = \frac{1}{l_{path}}\ln\frac{I_{in}}{I} \quad \text{(S6)}$$

The turbidity, τ measured in NTU by commercial turbidity meters is therefore expected to be proportional to absorbance A, measured by the UV-Vis spectrophotometer. The turbidity is related to droplet size distribution and number density of droplets via theory of light scattering from spherical particles as[16]:

$$\tau = \sum_{i=1}^{N_B}\frac{\pi d_{mi}^2}{4}N_i K_i \quad \text{(S7)}$$

Where N_i is concentration of number of droplets of bin i (number/m^3), K_i is scattering coefficient for droplets of size d_{mi}. The concentration of droplets is related to volume fraction of oil in measurement path (ε_O) as:

$$N_i = \frac{\varepsilon_O\, f(d_{mi})\Delta d_{mi}}{\frac{\pi d_{mi}^3}{6}} \quad \text{(S8)}$$

Substituting Equation (10) into Equation (11) leads to:

$$\tau = \frac{3\,\varepsilon_O}{2}\sum_{i=1}^{N_B}\frac{K_i\, f(d_{mi})\Delta d_{mi}}{d_{mi}} \quad \text{(S9)}$$

If the effective ratio of scattering coefficient and diameter is written as:

$$\frac{K_c}{d_c} = \sum_{i=1}^{N_B} \frac{K_i\, f(d_{mi}) \Delta d_{mi}}{d_{mi}} \qquad \text{(S10)}$$

Where K_c is an effective scattering coefficient and d_c is an effective characteristic droplet diameter. The scattering coefficient attains a value of 2 for droplets diameter much larger than the wavelength of light. Therefore, by setting the value of K_c to 2, Equation (S9) can be simplified as:

$$\tau = \frac{3\,\varepsilon_O}{d_{eff}} \qquad \text{(S11)}$$

S8. Energy consumption data for batch and continuous mode of emulsion production

The energy consumption values for both the mode of emulsion production compared to show that values are similar.

Table S6. Comparison of energy consumption for batch (B) and continuous mode of emulsion production

Q/q_{net}	α_o=0.15, B [kJ/kg]	α_o=0.15, C [kJ/kg]	α_o=0.30, B [kJ/kg]	α_o=0.30, C [kJ/kg]	α_o=0.45, B [kJ/kg]	α_o=0.45, C [kJ/kg]
1	0.20	0.195	0.205	0.197	0.208	0.2
5	1.01	0.973	1.026	0.985	1.040	1.0
20	4.05	3.892	4.105	3.942	4.159	4.0
100	20.26	19.46	20.523	19.708	20.796	19.96
